# A prognostic model, including the EBV status of tumor cells, for primary gastric diffuse large B‐cell lymphoma in the rituximab era

**DOI:** 10.1002/cam4.1595

**Published:** 2018-06-01

**Authors:** Eri Ishikawa, Tsutomu Tanaka, Kazuyuki Shimada, Kei Kohno, Akira Satou, Ahmed E. Eladl, Ayako Sakakibara, Kazuhiro Furukawa, Kohei Funasaka, Ryoji Miyahara, Masanao Nakamura, Hidemi Goto, Shigeo Nakamura, Seiichi Kato, Yoshiki Hirooka

**Affiliations:** ^1^ Department of Gastroenterology and Hepatology Nagoya University Graduate School of Medicine Nagoya Japan; ^2^ Department of Pathology and Laboratory Medicine Nagoya University Hospital Nagoya Japan; ^3^ Department of Endoscopy Aichi Cancer Center Hospital Nagoya Japan; ^4^ Department of Hematology and Oncology Nagoya University Graduate School of Medicine Nagoya Japan; ^5^ Department of Pathology Aichi Medical University Hospital Nagakute Japan; ^6^ Department of Pathology Faculty of Medicine Mansoura University Mansoura Egypt; ^7^ Department of Endoscopy Nagoya University Hospital Nagoya Japan; ^8^ Department of Pathology and Molecular Diagnostics Aichi Cancer Center Hospital Nagoya Japan

**Keywords:** diffuse large B‐cell lymphoma, Epstein‐Barr virus, gastric lymphoma, PD‐L1, rituximab

## Abstract

EBV‐positive diffuse large B‐cell lymphoma (DLBCL), not otherwise specified (NOS), often affects the gastrointestinal tract. However, the prognostic significance of EBV associated with primary gastric DLBCL (gDLBCL) has not been established. This retrospective study included 240 patients with primary gDLBCL, diagnosed between 1995 and 2015. Tumor specimens were analyzed with EBER in situ hybridization. In 25 (10%) cases, tumor cells harbored EBV. The EBV ^+^ group more frequently exhibited programmed death‐ligand 1 (PD‐L1) expression in microenvironment immune cells, but not tumor cells, compared to the EBV ^−^ group (86% vs 43%, *P *= .006). Among 156 patients that received rituximab‐containing chemotherapy, the EBV ^+^ group had a significantly worse overall survival (OS) than the EBV ^−^ group (*P *=* *.0029). Multivariate analyses identified 3 independent adverse prognostic factors of OS: multiple gastric lesions (*P *=* *.002), EBER positivity (*P *=* *.003), and B symptoms (*P *=* *.018). These factors were combined to develop a gDLBCL prognostic (gDLP) model that significantly stratified the patients into 3 distinct risk groups (Scores: good = 0, intermediate = 1, and poor = 2/3, *P *<* *.0001) with 5‐year OS rates of 100%, 81%, and 39%, respectively. Patients with EBV ^+^ gDLBCL commonly exhibited microenvironmental PD‐L1 expression and showed a significantly worse prognosis than subjects with EBV ^−^ gDLBCL. Our gDLP model, which included EBV ^+^ tumor cells, provided good predictions of clinical outcome and may be useful for selecting patients in trials in the immune‐oncology era.

## 1 INTRODUCTION

Within the gastrointestinal (GI) tract, the stomach is the most common site of non‐Hodgkin lymphoma (NHL). About half the gastric lymphoma cases are diagnosed as diffuse large B‐cell lymphoma (DLBCL),^1^, ^2^ which is a clinically, pathologically, and molecularly heterogeneous entity.^3^, ^4^, ^5^ Epstein‐Barr virus (EBV), a member of the herpes virus family, is implicated in numerous reactive and neoplastic processes of the immune system.^6^, ^7^ EBV‐positive (EBV^+^) DLBCL, not otherwise specified (NOS), often shows an aggressive clinical course with frequent extranodal disease that affects the GI tract.^8^, ^9^, ^10^ Previous reports have detected EBV in 5%‐15% of patients with DLBCL.^8^, ^11^, ^12^ In the current rituximab era, the prognostic significance of this finding is controversial.^9^, ^13^, ^14^, ^15^ Therefore, the clinicopathological and prognostic significance of EBV associated with primary gastric DLBCL (gDLBCL) has not been well established.

Targeted therapies that use antibodies against programmed cell death 1 (PD‐1) and its ligand (PD‐L1) have recently shown great promise in treating various malignancies, including relapsed or refractory DLBCL.^16^, ^17^ Overexpression of PD‐L1 on either DLBCL cells or tumor‐infiltrating immune cells has been associated with tumor cells that harbor EBV.^18^ Additionally, clinical trial studies have reported that the immune microenvironment of tumors generally correlates with the response rate to anti‐PD‐1/PD‐L1 therapies.^19^

In patients with gDLBCL, outcome is typically predicted with the Lugano staging system for GI tract lymphoma and the International Prognostic Index (IPI) for aggressive NHL.^20^, ^21^ However, these classification systems were devised in the pre‐rituximab era; that is, before rituximab was routinely added to chemotherapy, due to its positive impact in patients with DLBCL.^22^ With the advent of novel immune‐based therapies, there is a need to find alternative methods for identifying candidate targets for therapeutic immune checkpoint inhibitors.

In this study, we investigated 240 cases of primary gDLBCL to shed light on EBV‐harboring tumor cells. We aimed to develop a gDLBCL‐specific model designed for the rituximab era, which could be useful in selecting patients for clinical trials for immune‐oncology therapeutics.

## 2 MATERIALS AND METHODS

### 2.1 Patient selection

This retrospective study included data on 240 patients with primary gDLBCL diagnosed between 1995 and 2015 at Nagoya University Hospital and 30 affiliated institutions. All clinical and laboratory data were obtained from the medical records at each institution. The diagnosis was established according to histopathologic and immunohistochemical criteria, based on the 2017 WHO classification system. All cases satisfied the criteria for primary gastrointestinal lymphoma as defined by Lewin et al.^23^ The best method for discriminating primary gastric DLBCL from systemic DLBCL involving the stomach is not clear. Lymphoma at the stomach was considered primary if the main bulk of disease is located in the stomach. The clinical stage was evaluated according to the Lugano classification for gastrointestinal NHL.^20^ The endoscopic appearance of the tumors was determined based on the classification of Watanabe et al,^24^ with some modifications; that is, we added the following tumor classification features: superficial‐spreading type, mass‐forming type, diffuse‐infiltrating type, and mixed type. The mass‐forming tumor type was further classified into ulcerated and polypoid types. Multiple gastric lesions were defined as the presence of 2 or more lesions in the stomach, with adjacent, non‐neoplastic mucosa. The endoscopic findings were evaluated by 3 experienced endoscopists (E.I., K.F., and K.F.). All cases were reviewed by E.I., S.K., and S.N., and the consensus for diagnosis was reached with a multi‐headed microscope. The study was approved by the Institutional Review Board of Nagoya University.

### 2.2 Immunohistochemistry and in situ hybridization studies

Tissue samples were fixed in 10% formalin and embedded in paraffin. The samples were cut into 5‐μm‐thick sections and then stained with hematoxylin and eosin. The monoclonal antibodies used for immunohistochemistry are listed in the Table S1.

Cases were designated as either a germinal center B cell (GCB) or a non‐GCB immunophenotype, based on the Hans criteria.^4^ Lymphoid cell staining was considered positive for PD‐L1, when ≥5% of the neoplastic lymphoid cells showed moderate or strong membrane staining with a PD‐L1‐specific antibody (clone SP142). A case was considered positive for PD‐L1 in the microenvironment when, among the total tissue cellularity, ≥20% comprised nonmalignant cells with moderate or strong membrane or cytoplasmic PD‐L1‐specific staining. The threshold used here is comparable to that from a prior publication involving the same clone antibody.^25^, ^26^ To verify/falsify PD‐L1 expression on tumor and nonmalignant large lymphoid cells in controversial cases, PD‐L1/PAX5 and PD‐L1/CD68 double staining were applied and performed analogously as described elsewhere.^18^ All cases were tested for EBV‐encoded small RNA (EBER) with in situ hybridization (ISH), as described previously.^8^ Cases were considered EBER‐positive when nuclear expression of EBER was observed in ≥80% of tumor cells. Immunohistochemical double staining for EBER‐ISH and CD3 or CD79a was performed in selected cases.

### 2.3 Statistical analysis

Correlations between 2 groups were determined with the Fisher exact test and Mann‐Whitney *U* test. Survival distributions were estimated with the Kaplan‐Meier method, and groups were compared with the log‐rank test. Univariate Cox regression analyses were performed to assess the effects of prognostic factors. Only variables that were statistically significant in the univariate analysis were subsequently evaluated in the multivariate analysis. The multivariate analysis was performed with a forward/backward stepwise method, and *P *<* *.05 was the threshold for inclusion in the model. All statistical analyses were performed with the STATA software package, version 15 (Stata Corporation, College Station, TX, USA).

## 3 RESULTS

### 3.1 Clinicopathological characteristics of primary gastric DLBCL

The study cohort consisted of 240 patients with gDLBCL, including 136 males and 104 females (male: female ratio = 1.3:1), with a median age of 67 years (range, 32‐89 years). Clinical and treatment information was available for 239 patients. Their clinicopathologic and endoscopic data are summarized in Table 1. Of these, 129 patients (54%) were in Lugano stage I/II1 and 110 patients (46%) were in Lugano stage II2/IIE/IV; 69 patients (29%) had an IPI of high‐intermediate/high (HI/H); 57 patients (24%) exhibited B symptoms; and 1 patient (0.4%) had perforation during rituximab‐containing chemotherapy. Among the patients evaluated endoscopically (n = 128), 3 tumors (2%) were the superficial‐spreading type, 111 (87%) were the mass‐forming type, 2 (2%) were the diffuse‐infiltrating type, and 12 (9%) were the mixed type. In addition, 42 patients (33%) had multiple gastric lesions (Figure S1).

**Table 1:** Clinicopathological characteristics of patients with primary gastric DLBCL

Characteristics	Total (n=240)		EBV‐positive (n=25)		EBV‐negative (n=215)		*P* a
	No.	%	No.	%	No.	%	
Sex (male/female)	136/104	1.3	12/13	0.9	124/91	1.4	.39
Age (y), median (range)	67 (32‐89)		69 (37‐85)		67 (32‐89)		.90
Age >60 y	170/240	71	18/25	72	152/215	71	1.00
Abdominal pain	39/81	48	2/7	29	37/74	50	.43
Perforation	1/230	0.4	0/22	0	1/208	0.5	1.00
PS 2‐4	28/239	12	2/25	8	26/214	12	.75
Lugano stage II2/IIE/IV	110/239	46	13/25	52	97/214	45	.76
Serum LDH >normal	87/237	37	11/25	44	76/212	36	.53
sIL‐2R ≥1000 U/mL	101/211	48	9/19	47	92/192	48	1.00
Extranodal involvement >1 site	61/239	26	4/25	16	57/214	27	.34
IPI High‐int, High	69/237	29	7/25	28	62/212	29	1.00
B symptoms present	57/239	24	9/25	36	48/214	22	.14
*Helicobacter pylori* infection	49/72	68	5/7	71	44/65	68	1.00
Endoscopic appearance							
Superficial‐spreading type	3/128	2	0/21	0	3/107	3	1.00
Mass‐forming type	111/128	87	18/21	86	93/107	87	1.00
Ulcerated type	93/128	73	13/21	62	80/107	75	.28
Polypoid type	18/128	14	5/21	24	13/107	12	.18
Diffuse‐infiltrating type	2/128	2	2/21	10	0/107	0	.026
Mixed type	12/128	9	1/21	5	11/107	10	.69
Multiple gastric lesions	42/128	33	9/21	43	33/107	31	.32
Bulky mass present	34/240	14	3/25	12	31/215	14	1.00
Immunophenotype							
CD5	7/233	3	1/23	4	6/210	3	.52
CD10	58/234	25	7/23	30	51/211	24	.61
CD20	231/240	96	22/25	88	209/215	97	.055
CD30	4/52	8	1/13	8	3/39	8	1.00
BCL‐2	95/212	45	12/22	55	83/190	44	.37
BCL‐6	139/228	61	12/23	52	127/205	62	.38
MUM1	163/228	72	17/23	74	146/205	71	1.00
nPD‐L1 (≥5%)	0/54	0	0/14	0	0/40	0	—
miPD‐L1 (≥20%)	29/54	54	12/14	86	17/40	43	.006
Non‐GCB immunophenotype	145/231	63	16/24	67	129/207	62	.82
Treatment							
R‐containing CTx	156/239	65	12/25	48	144/214	67	.075
R‐CTx	91/156	58	8/12	67	83/144	58	.76
R‐CTx + RT	55/156	35	3/12	25	52/144	36	.54
R‐CTx + Surgery	9/156	6	0/12	0	9/144	6	1.00
R‐CTx + Surgery + RT	1/156	0.6	1/12	8	0/144	0	.077
No. of cycles, median (range)	5 (1‐8)		3.5 (2‐8)		5 (1‐8)		.22
No treatment	5/239	2	3/25	12	2/214	1	.009
Therapeutic response (R‐containing CTx)							
CR	135/156	87	9/12	75	126/144	88	.21
PR	13/156	8	1/12	8	12/144	8	1.00
SD	1/156	1	1/12	8	0/144	0	.077
PD	7/156	4	1/12	8	6/144	4	.44

CR, complete remission; CTx, chemotherapy; GCB, germinal center B‐cell; High‐int, high‐intermediate; IPI, International Prognostic Index; LDH, lactate dehydrogenase; miPD‐L1, microenvironmental programmed cell death ligand 1; nPD‐L1, neoplastic programmed cell death ligand 1; PD, progressive disease; PR, partial remission; PS, performance status; sIL‐2R, soluble interleukin‐2 receptors; R, rituximab; RT, radiotherapy; SD, stable disease.

a *P* value are for the comparison of EBV‐positive and EBV‐negative primary gastric DLBCL patients.

Among the 240 patients enrolled in this study, all the punch biopsies (n = 237, 99%) and surgically resected specimens (n = 3, 1%) exhibited a predominant proliferation of medium‐to‐large lymphoid cells without evidence of concomitant low‐grade lesions. Paraffin‐section immunohistochemistry specimens were available for all patients. Of these 240 specimens, 231 (96%) showed CD20 positivity on tumor cells. The remaining CD20‐negative cases (n = 9, 4%) were positive for CD79a. Twenty‐five (10%) cases showed EBV harboring on ≥80% of their tumor cells by EBER‐ISH, but EBV was not detected in the background cells. Of 54 cases examined for PD‐L1, 29 (54%) showed positive PD‐L1 signals; these signals were detected in microenvironmental immune cells, but not in tumor cells.

### 3.2 Treatment of primary gastric DLBCL

In the proportion of patients principally treated with chemotherapy (n = 148), with no surgical resection or irradiation, overall survival (OS) was significantly different between the subgroups treated with or without rituximab (n = 91 and 57, respectively). Patients that received rituximab showed a more favorable course (*P *<* *.001). Based on this finding, we limited subsequent survival analyses to the group of patients that received rituximab‐containing chemotherapy.

Of 239 patients with primary gDLBCL, 156 (65%) received multi‐agent chemotherapy combined with rituximab. Of these, 91 (58%) received only rituximab‐containing chemotherapy, and 55 (35%) received additional irradiation. Of the 156 patients that received chemotherapy, 150 (96%) received anthracycline‐based chemotherapy, including 146 (94%) that also received CHOP (cyclophosphamide, doxorubicin, vincristine, prednisolone).

### 3.3 Treatment response and prognosis of primary gastric DLBCL in the rituximab era

Among 156 patients with gDLBCL that received rituximab‐containing chemotherapy, 135 (87%) achieved a complete response (CR) and 7 (4%) developed progressive disease (PD). In this group, the 5‐year OS and progression‐free survival (PFS) rates were 83% and 75%, respectively, with a median follow‐up of 60 months (range, 4‐141 months). The 5‐year OS rates for patients with Lugano stages I (n = 54), II1 (n = 37), II2 (n = 12), IIE (n = 8), and IV (n = 45) were 94%, 100%, 61%, 63%, and 66%, respectively. On the other hand, the 5‐year OS rates for patients with IPI values of low (n = 87), low‐intermediate (n = 24), high‐intermediate (n = 26), and high (n = 19) were 94%, 77%, 78%, and 45%, respectively. No statistically significant difference was noted in OS between cases with positive and negative detection of PD‐L1 in the microenvironment.

### 3.4 Clinicopathological characteristics of EBV^+^ primary gastric DLBCL

Twenty‐five (10%) of 240 cases with gDLBCL showed EBV harboring on ≥80% of their tumors by EBER‐ISH. In 22 of 25 EBV^+^ gDLBCL cases with available FFPE sections, LMP1 and EBNA2 were found in 15 (68%) and 12 (55%) of them, respectively. They were diagnosed as EBV latency II and III for 4 (18%) and 12 cases (55%), respectively, in our series. We found no significant difference in clinicopathological findings between the EBV^+^ and EBV^−^ gDLBCL subgroups, except for the endoscopic features and microenvironmental PD‐L1 expression (Table 1). Endoscopic findings showed that the EBV^+^ subgroup had a higher prevalence of the diffuse‐infiltrating tumor type (10%) than the EBV^−^ subgroup (0%, *P *=* *.026). Histological results showed that PD‐L1 was detected in microenvironmental immune cells at a significantly higher rate in the EBV^+^ gDLBCL group (n = 12/14, 86%) than in the EBV^−^ group (n = 17/40, 43%; *P *=* *.006; Figure S2).

### 3.5 Comparison of clinical courses in patients with EBV^+^ and EBV^−^ primary gastric DLBCL in the rituximab era

**Figure 1:**
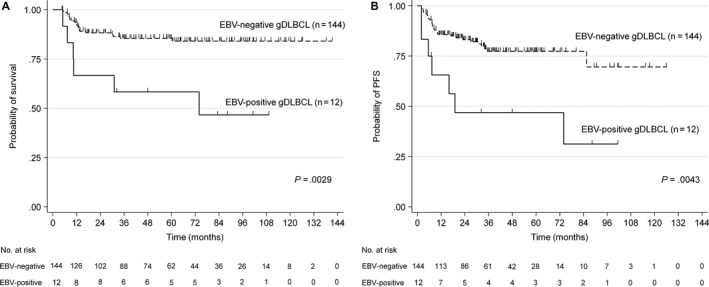
Survival analysis of patients with gDLBCL treated with rituximab‐containing chemotherapy. A, Overall survival; B, Progression‐free survival according to EBV status on tumor cells

We observed no significant difference in therapeutic response between the EBV^+^ and EBV^−^ subgroups. However, among the patients with gDLBCL that received rituximab‐containing chemotherapy, the OS and PFS rates were significantly worse in the EBV^+^ subgroup than in EBV^−^ subgroup (5‐year OS: 58% vs 84%; *P *=* *.0029; 5‐year PFS: 47% vs 77%; *P *=* *.0043; Figure 1).

### 3.6 Clinical course of EBV^+^ primary gastric DLBCL

**Figure 2:**
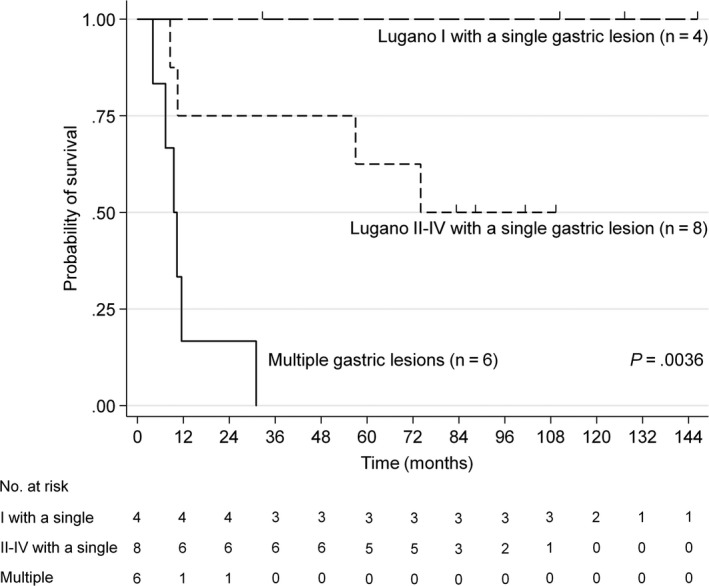
Overall survival according to the combination of Lugano stage classification and the number of gastric lesions in patients with EBV ^+^ gDLBCL patients treated with chemotherapy

Among patients with EBV^+^ gDLBCL principally treated with chemotherapy (n = 16), with no surgical resection or irradiation, there was no benefit from the addition of rituximab (*P *=* *.6068, Figure S3). Therefore, we performed a univariate analysis for further stratification of our patients with EBV^+^ gDLBCL that received chemotherapy (with or without rituximab) (n = 21). This analysis identified 3 factors associated with a poor prognosis: multiple gastric lesions (*P *=* *.0031), Lugano stage II2/IIE/IV (*P *=* *.0173), and high levels of sIL‐2R (*P *=* *.0434; Table 2). Based on this result, we stratified patients with EBV^+^ gDLBCL treated with chemotherapy into 3 groups by combination of Lugano stage classification and the number of gastric lesions: those with a single gastric lesion in Lugano stage I (n = 4), those with a single gastric lesion in Lugano stage II1/II2/IIE/IV (n = 8), and those with multiple gastric lesions (n = 6). The 5‐year OS rates for these 3 subgroups were 100%, 63%, and 0%, respectively. This classification significantly stratified patients with EBV^+^ gDLBCL by survival risk (*P *=* *.0036, Figure 2).

**Table 2:** Univariate analysis for OS in EBV‐positive primary gastric DLBCL (n = 21)

Variables		Univariate analysis	
		HR (95% CI)	*P*
Sex	Male	1.49 (0.46‐4.80)	.5029
Age	>50 y	1.96 (0.25‐15.2)	.5189
PS	2‐4	3.05 (0.61‐15.2)	.1734
Lugano stage	II2/IIE/IV	5.06 (1.33‐19.2)	.0173
Serum LDH	>Normal	3.05 (0.91‐40.3)	.0719
sIL‐2R	≥1000 U/mL	5.37 (1.05‐27.4)	.0434
Extranodal involvement	>1 site	2.02 (0.54‐7.65)	.2987
IPI	High‐int, High	1.86 (0.58‐5.99)	.2983
B symptoms	Present	1.72 (0.54‐5.49)	.3599
Endoscopic appearance	Ulcerated type	1.01 (0.26‐3.94)	.9845
Multiple gastric lesions	Present	11.9 (2.31‐61.6)	.0031
Bulky mass	Present	1.60 (0.34‐7.61)	.5534
Immunophenotype			
CD5	Positive	3.27 (0.38‐28.0)	.2798
CD30	Positive	2.12 (0.23‐19.2)	.5832
BCL‐2	Positive	1.51 (0.43‐5.31)	.5226
miPD‐L1 (≥20%)	Negative	1.15 (0.13‐10.4)	.9029
Pathological subtype	Non‐GCB immunophenotype	1.71 (0.46‐6.34)	.4220
R‐containing CTx	None	1.58 (0.51‐4.92)	.4277

CTx, chemotherapy; High‐int, high‐intermediate; IPI, International Prognostic Index; LDH, lactate dehydrogenase; miPD‐L1, microenvironmental programmed cell death ligand 1; non‐GCB, non‐germinal center B‐cell; OS, overall survival; PS, performance status; R, rituximab; sIL‐2R, soluble interleukin‐2 receptors.

### 3.7 Prognostic factors of primary gastric DLBCL in the rituximab era

A univariate analysis identified 9 prognostic factors for survival in patients with gDLBCL that received rituximab‐containing chemotherapy (Table 3). Based on these 9 factors, survival was unfavorable for patients with Lugano stage II2/IIE/IV (*P *<* *.0001), more than 1 site of extranodal involvement (*P *=* *.0001), high levels of serum LDH (*P *=* *.0002), IPI HI/H (*P *=* *.0002), multiple gastric lesions (*P *=* *.0004), high levels of soluble interleukin‐2 receptors (sIL‐2R ≥1000 U/mL, *P *=* *.0021), EBER positivity (*P *=* *.0029), B symptoms (*P *=* *.0066), and BCL‐2 positivity (*P *=* *.0250). A multivariate analysis indicated that the presence of multiple gastric lesions (*P *=* *.002), EBER positivity (*P *=* *.003), and B symptoms (*P *=* *.018) were the only independent adverse prognostic factors.

**Table 3:** Univariate and multivariate analysis for OS in primary gastric DLBCL in the rituximab era (n = 156)

Variables		Univariate analysis		Multivariate analysis^a^	
		HR (95% CI)	*P*	HR (95% CI)	*P*
Sex	Male	1.15 (0.52‐2.53)	.7336		
Age	>60 y	1.92 (0.73‐5.10)	.1888		
PS	2‐4	2.62 (0.99‐6.97)	.0534		
Lugano stage	II2/IIE/IV	9.39 (3.23‐27.3)	<.0001		
Serum LDH	>Normal	4.74 (2.16‐10.4)	.0002		
sIL‐2R	≥1000 U/mL	4.03 (1.65‐9.80)	.0021		
Extranodal involvement	>1 site	4.84 (2.22‐10.6)	.0001		
IPI	High‐int, High	4.54 (2.08‐9.94)	.0002		
B symptoms	Present	2.95 (1.35‐6.42)	.0066	4.01 (1.27‐12.6)	.018
*Helicobacter pylori* infection	Negative	3.20 (0.29‐35.4)	.3422		
Endoscopic appearance	Except mass‐forming type	1.48 (0.42‐5.17)	.5369		
Multiple gastric lesions	Present	7.63 (2.48‐23.4)	.0004	12.3 (2.59‐58.8)	.002
Bulky mass	Present	2.40 (0.96‐5.97)	.0606		
Immunophenotype					
CD5	Positive	1.23 (0.17‐9.15)	.8364		
BCL‐2	Positive	2.64 (1.13‐6.17)	.0250		
miPD‐L1 (≥20%)	Positive	1.11 (0.25‐4.99)	.8955		
EBER	Positive	4.01 (1.61‐10.0)	.0029	6.74 (1.92‐23.7)	.003
Pathological subtype	GCB immunophenotype	1.05 (0.46‐2.39)	.9133		

EBER, EBV‐encoded small RNA; High‐int, high‐intermediate; IPI, International Prognostic Index; LDH, lactate dehydrogenase; miPD‐L1, microenvironmental programmed cell death ligand 1; non‐GCB, non‐germinal center B‐cell; OS, overall survival; PS, performance status; sIL‐2R, soluble interleukin‐2 receptors.

a The variables included in multivariate analysis for OS were age, Lugano stage, serum LDH, sIL‐2R, extranodal involvement, B symptoms, multiple gastric lesions, BCL‐2, EBER.

### 3.8 Prognostic model of primary gastric DLBCL in the rituximab era

**Figure 3:**
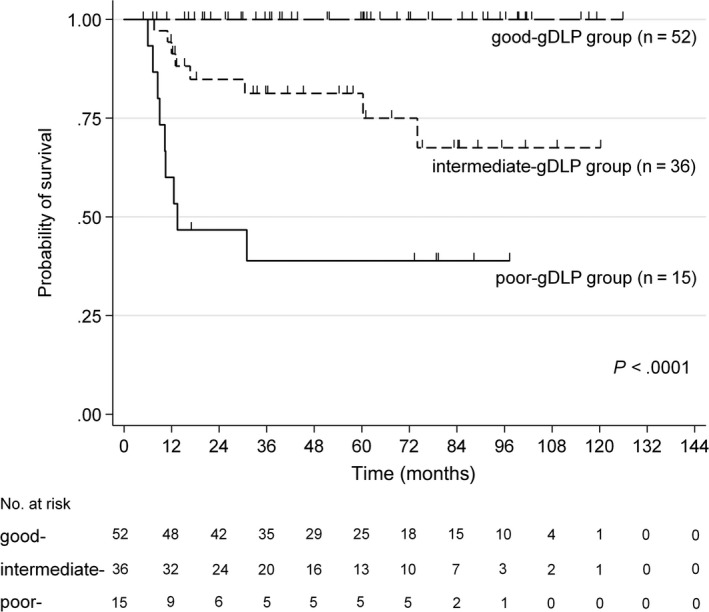
Overall survival of patients with gDLBCL treated with rituximab‐containing chemotherapy, predicted with gastric DLBCL prognostic (gDLP) model. This model was constructed by the assignment of patient risk scores (0, 1, 2, or 3) based on the number of risk factors present (EBER positivity, multiple gastric lesions, and/or B symptoms). The 3 risk groups were defined as good‐, intermediate‐, and poor‐gDLP, which corresponded to risk scores of 0, 1, and 2/3, respectively

We constructed a gDLBCL prognostic (gDLP) model by combining the 3 independent prognostic variables; that is, multiple gastric lesions, EBER positivity, and B symptoms. Patients were scored according to the following gDLP scores: 0 = no adverse factors (n = 52); 1 = positive for 1 factor (n = 36); 2 = positive for 2 factors (n = 14); and 3 = positive for 3 factors (n = 1). The 5‐year OS rates for these 4 groups were 100%, 81%, 42%, and 0%, respectively. Our prognostic model stratified the patients into gDLP groups with good (gDLP score 0 [n = 52]), intermediate (gDLP score 1 [n = 36]), and poor (gDLP score 2/3 [n = 15]) prognoses. In good‐gDLP group, 33 (63%) of 52 were treated with rituximab‐containing chemotherapy alone, 15 (29%) received additional irradiation, and 4 (8%) underwent surgical resection initially. On the other hand, in poor‐gDLP group, 13 (87%) of 15 received rituximab‐containing chemotherapy alone and two (13%) received additional irradiation. With this model, the patients with gDLBCL were accurately stratified by survival risk (*P *<* *.0001, Figure 3).

## 4 DISCUSSION

This study aimed to clarify the prognostic significance of EBV associated with gDLBCL and define the characteristics of patients with a poor prognosis to facilitate patient selection for clinical trials in the current era of immuno‐oncology. To that end, we analyzed 240 primary gDLBCL patients, of whom 156 had been treated with rituximab‐containing chemotherapy. Our results revealed that EBV^+^ gDLBCL occurred uncommonly, but it was often aggressive and life‐threatening. In addition, this disease featured high PD‐L1 expression in microenvironmental immune cells and relatively diverse in biologic behavior as shown in Figure 2. We developed a prognostic model that comprised 3 independent factors associated with adverse outcome: multiple gastric lesions, B symptoms, and the presence of EBV‐harboring tumor cells. This model could accurately stratify patients with primary gDLBCL into 3 distinct prognostic groups. Further studies are warranted to establish optimal strategies for treating EBV^+^ tumor cells in primary gDLBCL.

Previous studies have described clinicopathological features that served as prognostic indicators of poor clinical outcome in gDLBCL, including *Helicobacter pylori* negativity,^27^ an absence of gene translocation involving the immunoglobulin heavy‐chain,^28^ Lugano stage II2/IIE/IV, elevated serum LDH levels,^29^ and so on. Given the marked heterogeneity of DLBCL, a reliable prediction tool is vital for optimizing patient treatment. Here, we found that the presence of EBV^+^ tumor cells was an independent prognostic indicator for patients with gDLBCL. This finding supported our prior assertion that DLBCL of the elderly or age‐related EBV associated B‐cell lymphoproliferative disorders constitutes a distinct clinicopathologic entity in contrast with EBV^−^ DLBCLs, in which conventional chemotherapy has a limited efficacy for this disease.^8^ Recent studies have reported that EBV^+^ DLBCL also occurred sporadically in young patients. That finding led us to the nosological term, “EBV^+^ DLBCL, NOS,” in the 2017 WHO classification.^15^, ^30^ Our finding that EBV had a negative impact on gDLBCL was consistent with findings reported by Sato et al and Hong et al,^9^, ^15^ but was inconsistent with the findings of Ok et al^14^ and Ahn et al,^13^ which indicated an equivalent prognosis for patients with EBV^+^ and EBV^−^ DLBCL in the rituximab era. These controversial conclusions may be partly due to the heterogeneity of patients with DLBCL. This heterogeneity was exemplified by the diversity of the affected anatomical sites reported in different studies. In addition, the inclusion of cases with a relatively low percentage of EBV^+^ tumor cells limits firm conclusions regarding EBV^+^ DLBCL and it is dubious whether in such cases the virus represents a true pathogenic event. Indeed, the above‐mentioned 4 studies used thresholds as 30%, 20%, 10%, and 10%, respectively, to define EBV^+^ DLBCL. In this study, we adapted the threshold of 80% for the diagnosis of EBV^+^ gDLBCL, which is recommended in the 2017 WHO classification, to avoid the inclusion of cases in which EBV may be a bystander in non‐neoplastic cells.

Very few studies have analyzed the significance of the endoscopic appearance and the abundance of gastric lesions in gDLBCL. In our series, approximately 90% of cases involved the mass‐forming type of tumor, similar to the rate reported by Nakamura et al^28^ in patients with gDLBCL without mucosa‐associated lymphoid tissue lymphoma. On the other hand, multifocal lesions were reported to be associated with secondary gastric NHL.^31^ To our knowledge, the significance of multiple gastric lesions on the clinical outcome of patients with gDLBCL had not been fully elucidated previously. In this study, we showed that, in addition to tumor cells that harbored EBV, multiple gastric lesions were independently associated with a poor OS in gDLBCL.

The IPI, which incorporates 5 clinical variables, remains a valuable prognostic tool for DLBCL in the rituximab era.^32^ The Lugano classification, which emphasizes the distribution of nodal and extranodal disease sites and the extension to adjacent organs, was proposed for GI tract lymphoma.^20^ However, due to tumor heterogeneity, risk‐stratification specific to gDLBCL has not been elucidated. In the present study, the Lugano classification distinguished 5 separate groups with 5‐year OS rates, ranging from 61% to 100%. In contrast, the IPI segregated patients into 4 outcome groups with 5‐year OS ranging from 45% to 94%. Based on our findings, we proposed a new gDLP model, which integrated 3 clinical parameters: multiple gastric lesions (*P *=* *.002), EBER positivity (*P *=* *.003), and B symptoms (*P *=* *.018). This model succeeded in identifying a low‐risk gDLP group with no adverse factors and an extremely favorable outcome in the rituximab era. In this group, 100% of patients remained alive after a median follow‐up of 56 months (range 5‐126 months). In contrast, patients in the high‐risk gDLP group, which consisted of 5 patients with EBV^+^ and 10 with EBV^−^ gDLBCL, was shown to have the most aggressive disease, with a 5‐year OS rate of only 39%.

Notably, among the 18 cases with EBV^+^ gDLBCL treated with chemotherapy, 6 had multiple gastric lesions. Aggressive disease in these 6 patients resulted in death within 3 years of diagnosis. Recent studies have shown that, blocking the interaction of PD‐1 with its ligands, PD‐L1 and PD‐L2, led to impressive antitumor responses and clinical benefit in a subset of patients,^33^ including those with relapsed and refractory DLBCL.^16^, ^17^ However, predicting tumor responses to PD‐1 blockade remains a major challenge. Some studies reported that responses to PD‐1/PD‐L1 blockade immunotherapies were observed in patients with PD‐L1 expression in tumor‐infiltrating immune cells.^19^ In addition, Kiyasu et al^18^ showed a close association between PD‐L1 expression in microenvironmental immune cells and EBV^+^ tumor cells, consistent with our results. Given those findings, we believe that patients with EBV^+^ gDLBCL represent good candidates for future clinical trial, when they show significant PD‐L1 expression in microenvironmental immune cells, and particularly, when multiple gastric lesions are present, due to their dismal prognosis.

In contrast to those patients, we identified 4 EBV^+^ cases with a single gastric lesion in Lugano stage I that displayed a plateau in the survival curve after diagnosis. The combination of these clinical features evoked the possibility of an EBV^+^ mucocutaneous ulcer (EBVMCU), characterized by indolent behavior and a self‐limited clinical course. However, in our series, 2 of these cases had polypoid tumors, and the remaining 2 cases had large ulcerated tumors, but unlike the superficial lesion commonly observed for EBVMCU. In the original report by Dojcinov et al,^34^ EBVMCU was shown to affect the esophagus, colon, and rectum, but not the stomach. Nevertheless, the favorable clinical course we observed for patients with a single gastric lesion in Lugano stage I suggested that this condition might warrant special recognition as a separate class from the other EBV^+^ gDLBCL cases.

In the present study, of 54 gDLBCL cases examined, all were negative for PD‐L1 in tumor cells, including 14 patients with EBV^+^ gDLCBL. This finding was considerably different from findings in previous studies, where PD‐L1 expression in tumor cells was observed in 26% of DLBCL, NOS cases examined with immunohistochemistry.^18^ The frequent up‐regulation of PD‐L1 was also shown in EBV^+^ post‐transplant lymphoproliferative disorders and EBV^+^ DLBCL.^30^, ^35^, ^36^ EBV was reported to provide an intrinsic signal to augment PD‐L1 expression through EBV‐LMP1 increasing PD‐L1 promoter and enhancer activity.^35^ In this study, none of EBV^+^ gDLBCL cases expressed PD‐L1 on tumor cells, although LMP1 was positive in 7 of 14 EBV^+^ cases evaluated. One potential explanation for these discrepancies could be the antibodies used. For example, Kataoka et al^37^ reported that a PD‐L1 3′‐UTR disruption caused a lack of PD‐L1 detection in virus‐related tumors that were probed with the SP142 antibody, which was directed against the C‐terminal domain. Therefore, we conducted a preliminary study to compare our results with results obtained with the E1J2J antibody, which is directed against the N‐terminal domain (n = 9 cases of EBV^+^ gDLBCL). However, again, we did not detect PD‐L1 expression on tumor cells of those patients. Another potential explanation for these discrepancies could be that our PD‐L1 staining was performed on small endoscopic biopsy specimens, in all 54 gDLBCL cases; thus, the small specimen might not have been representative of the PD‐L1 expression in a whole tumor. The other potential explanation for these discrepancies could be the difference among the primary sites of involvement (gastric site vs nodal). This issue should be examined further in the future studies.

In summary, our prognostic model, which included EBV^+^ tumor cells, multiple gastric lesions, and B symptoms, provided accurate definitions of 3 prognostic groups, including 1 group with the worst prognosis. In addition, EBV^+^ gDLBCL featured PD‐L1 expression in microenvironmental immune cells and those with multiple gastric lesions were most likely to have highly aggressive disease. We recommend that EBV should be routinely examined in patients with gDLBCL to provide better assessments of prognosis and better predictions of the therapeutic effects of immune‐oncology. This approach might facilitate patient selection in the future clinical trials. Our results warrant further validation in future studies.

## CONFLICT OF INTERESTS

The authors have no significant relationships with or financial interests in any commercial companies pertaining to this article.
