# Supplementary figures and images for: A prognostic model, including the EBV status of tumor cells, for primary gastric diffuse large B‐cell lymphoma in the rituximab era
Source: Cancer Med. 2018 Jun 1;7(7):3510–20. doi: 10.1002/cam4.1595 (PMC6051208; doi:10.1002/cam4.1595)

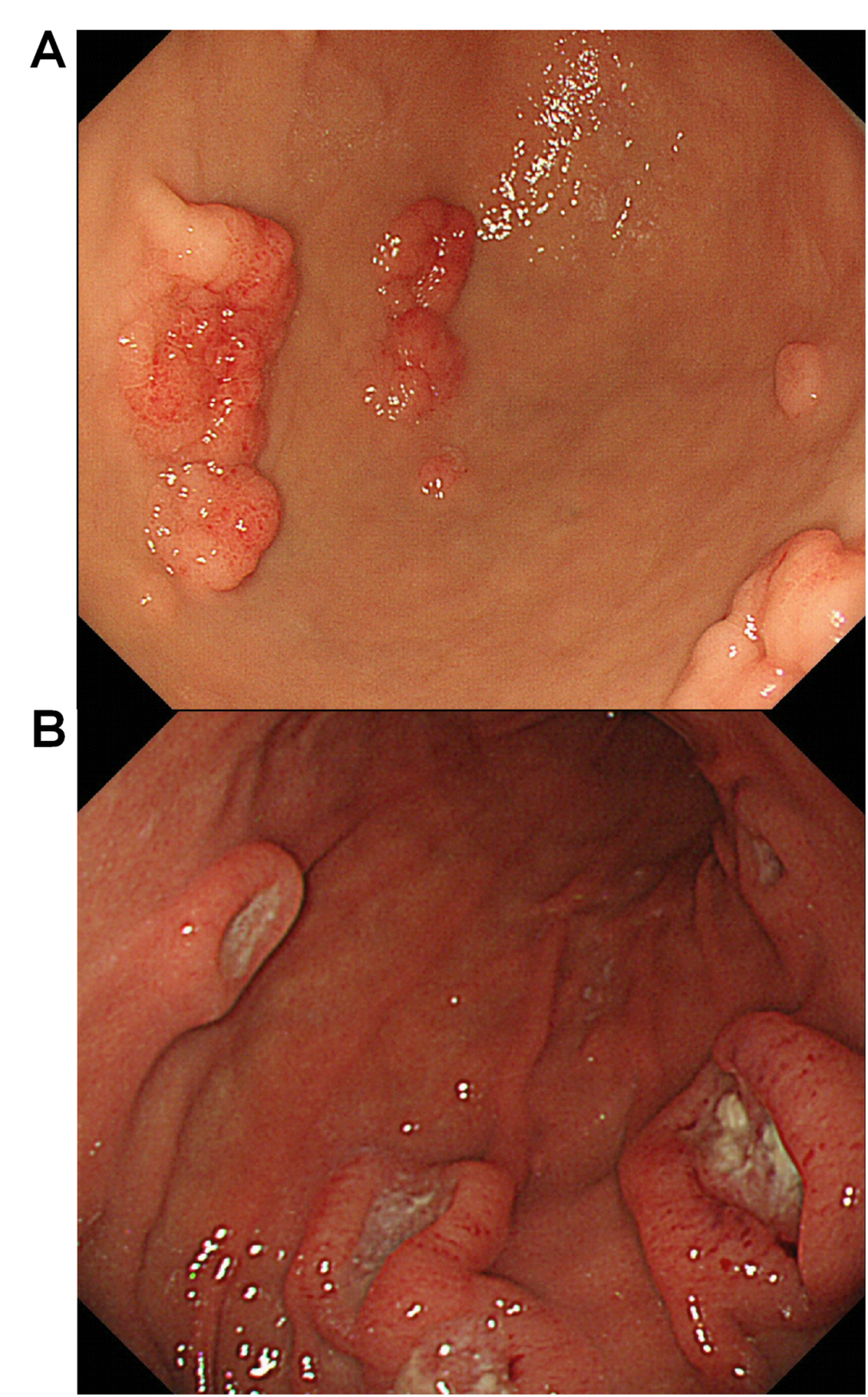

Supplement: Supplementary file 1 [file CAM4-7-3510-s001.tif]

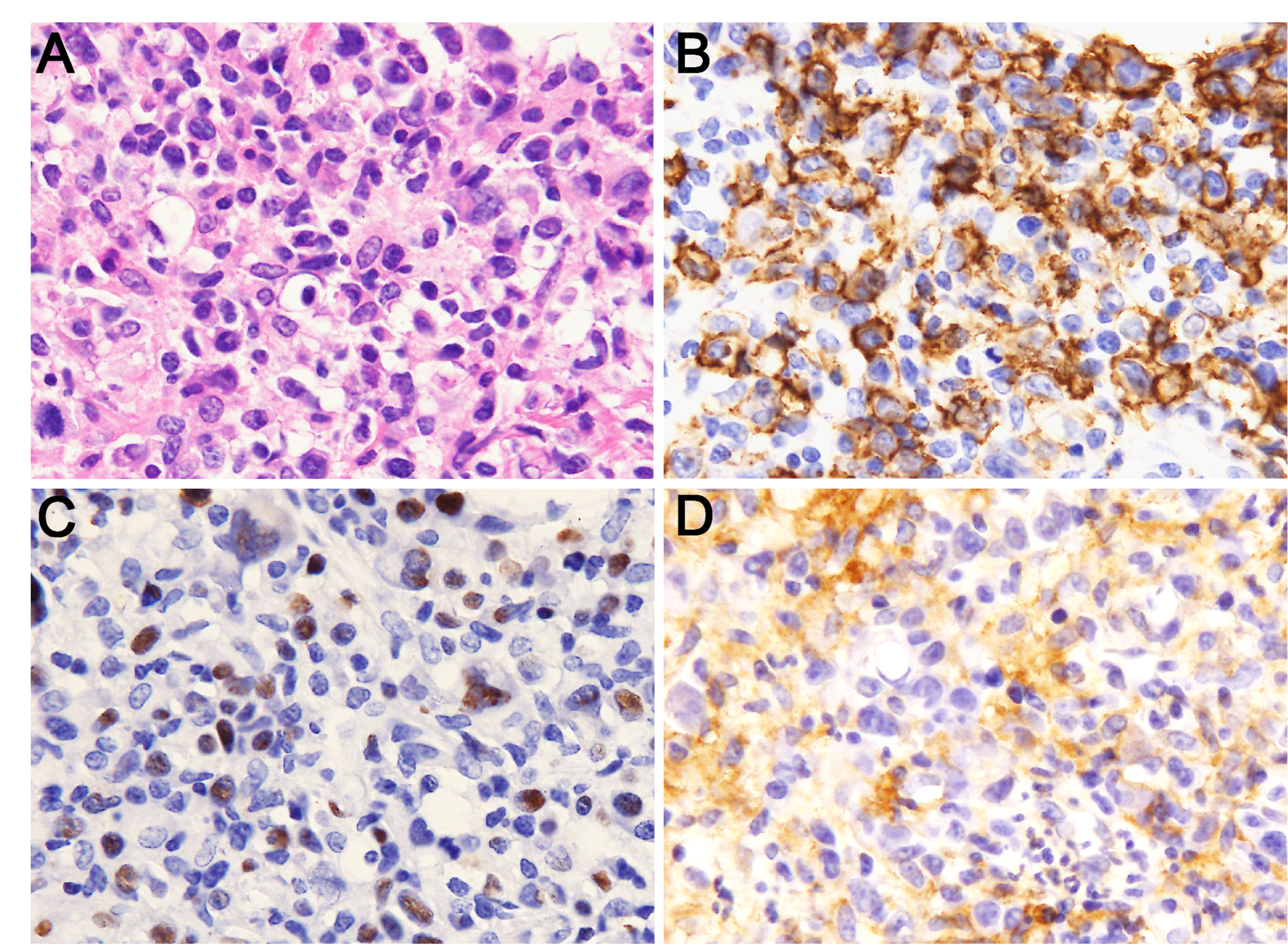

Supplement: Supplementary file 2 [file CAM4-7-3510-s002.tif]

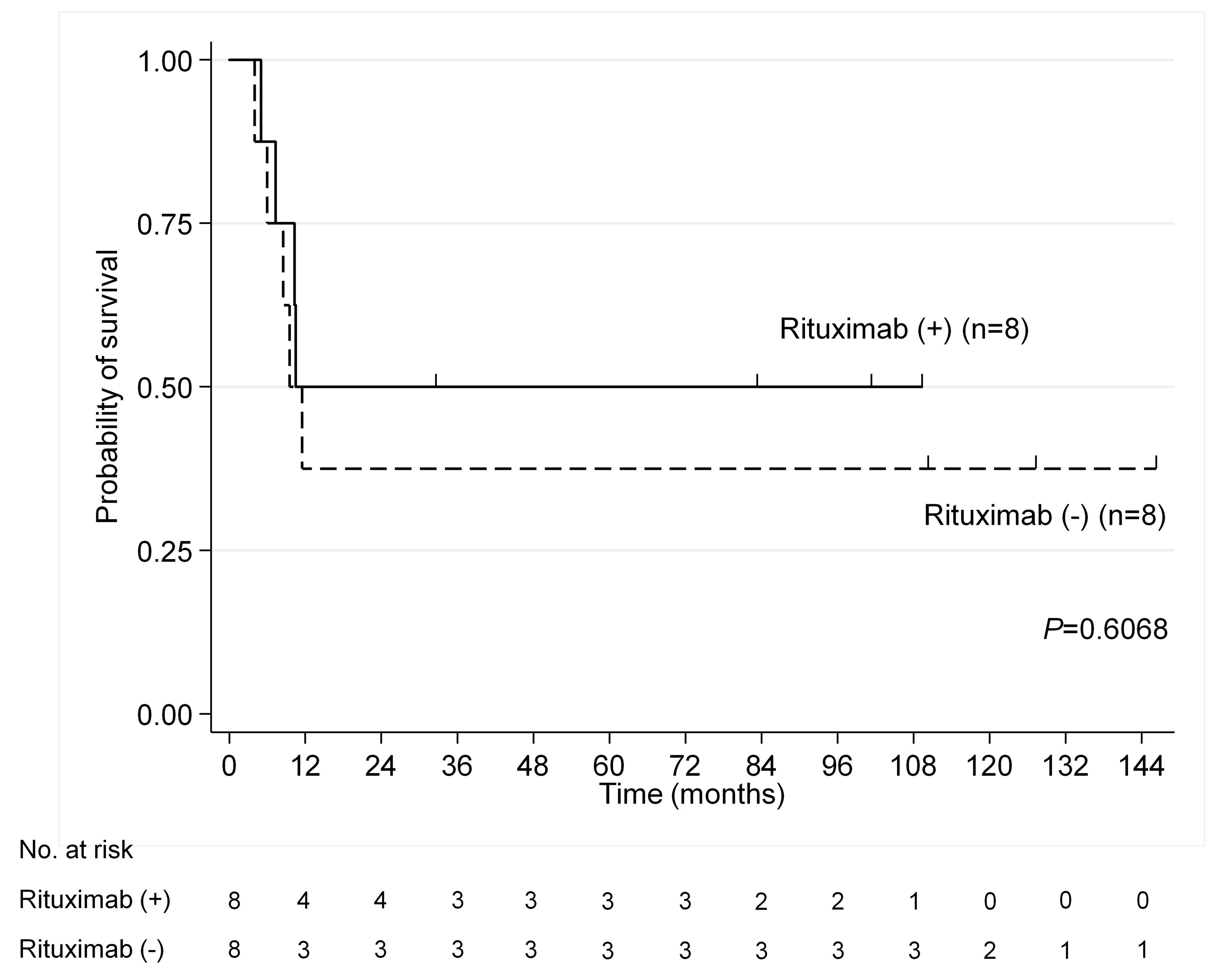

Supplement: Supplementary file 3 [file CAM4-7-3510-s003.tif]
