# Supplementary material for: A prognostic model, including the EBV status of tumor cells, for primary gastric diffuse large B‐cell lymphoma in the rituximab era
Source: Cancer Med. 2018 Jun 1;7(7):3510–20. doi: 10.1002/cam4.1595 (PMC6051208; doi:10.1002/cam4.1595)
Supplement: Supplementary file 4 [file CAM4-7-3510-s004.docx]

| **TABLE S1. Antibodies used in the immunophenotypic analysis of gastric DLBCL** | | | |
| --- | --- | --- | --- |
| Antigen | Clone | Dilution | Source |
| CD3 | F7.238 | 1:30 | Dako |
| CD10 | 56C6 | 1:40 | Dako |
| CD20 | L26 | 1:1 | Dako |
| CD30 | Ber-H2 | 1:20 | Dako |
| CD79a | JCB117 | 1:80 | Dako |
| Bcl-2 | l24 | 1:80 | Dako |
| Bcl-6 | LN22 | 1:20 | Novocastra |
| MUM1/IRF4 | MUM1P | 1:200 | Santa Cruz Biotechnology |
| PD-L1 | SP142 | 1:50 | Spring Bioscience |
| PD-L1 | E1J2J | 1:50 | Cell Signaling Technology |
| LMP1 | C-S.1-4 | 1:100 | Dako |
| EBNA2 | PE2 | 1:50 | Dako |
