# Supplementary material for: A prognostic model, including the EBV status of tumor cells, for primary gastric diffuse large B‐cell lymphoma in the rituximab era
Source: Cancer Med. 2018 Jun 1;7(7):3510–20. doi: 10.1002/cam4.1595 (PMC6051208; doi:10.1002/cam4.1595)
Supplement: Supplementary file 5 [file CAM4-7-3510-s005.docx]

**Supporting Figure legends**

Figure S1. Endoscopic appearance of lesions in patients with gDLBCL and multiple gastric lesions. (A) Multiple polypoid mass lesions on the greater curvature of the gastric body (mass-forming type [polypoid type]). (B) Multiple tumor masses with a central ulceration on the greater curvature of the gastric body (mass-forming type [ulcerated type]).

Figure S2. EBV^+^ gDLBCL histology. Specimens from patients with EBV^+^ gDLBCL showed (A) diffuse lymphoid proliferation of medium to large cells; hematoxylin and eosin stain. The tumor cells were positive for (B) CD20 and (C) EBER-ISH. (D) PD-L1 was expressed in microenvironmental immune cells.

Figure S3. Overall survival according to treatment with or without rituximab in EBV^+^ gDLBCL patients principally treated with chemotherapy alone (n=16).
